# Neuroinflammation in Autism: Plausible Role of Maternal Inflammation, Dietary Omega 3, and Microbiota

**DOI:** 10.1155/2016/3597209

**Published:** 2016-10-20

**Authors:** Charlotte Madore, Quentin Leyrolle, Chloé Lacabanne, Anouk Benmamar-Badel, Corinne Joffre, Agnes Nadjar, Sophie Layé

**Affiliations:** ^1^Ann Romney Center for Neurologic Diseases, Department of Neurology, Brigham and Women's Hospital, Harvard Medical School, Boston, MA, USA; ^2^Nutrition et Neurobiologie Intégrée, UMR 1286, INRA, 33000 Bordeaux, France; ^3^Nutrition et Neurobiologie Intégrée, UMR 1286, Bordeaux University, 33000 Bordeaux, France; ^4^Inserm, U1141, Hôpital Robert Debré, Paris, France; ^5^Université Paris Diderot, Sorbonne Paris Cité, Paris, France; ^6^Department of Psychiatry, McGill University, Montreal, QC, Canada; ^7^Douglas Mental Health University Institute, Montreal, QC, Canada; ^8^Département de Biologie, École Normale Supérieure de Lyon, Université de Lyon, UCB Lyon 1, Lyon, France; ^9^OptiNutriBrain International Associated Laboratory (NutriNeuro France-INAF Canada), Bordeaux, France

## Abstract

Several genetic causes of autism spectrum disorder (ASD) have been identified. However, more recent work has highlighted that certain environmental exposures early in life may also account for some cases of autism. Environmental insults during pregnancy, such as infection or malnutrition, seem to dramatically impact brain development. Maternal viral or bacterial infections have been characterized as disruptors of brain shaping, even if their underlying mechanisms are not yet fully understood. Poor nutritional diversity, as well as nutrient deficiency, is strongly associated with neurodevelopmental disorders in children. For instance, imbalanced levels of essential fatty acids, and especially polyunsaturated fatty acids (PUFAs), are observed in patients with ASD and other neurodevelopmental disorders (e.g., attention deficit hyperactivity disorder (ADHD) and schizophrenia). Interestingly, PUFAs, and specifically n-3 PUFAs, are powerful immunomodulators that exert anti-inflammatory properties. These prenatal dietary and immunologic factors not only impact the fetal brain, but also affect the microbiota. Recent work suggests that the microbiota could be the missing link between environmental insults in prenatal life and future neurodevelopmental disorders. As both nutrition and inflammation can massively affect the microbiota, we discuss here how understanding the crosstalk between these three actors could provide a promising framework to better elucidate ASD etiology.

## 1. Introduction

Autism is a complex neurodevelopmental condition whose different forms are described in DSM-V as autism spectrum disorder (ASD). ASD affects almost 1 in 100 children [1] and is characterized, in varying degrees, by deficits in verbal and nonverbal communication, and is associated with repetitive behaviors [2]. Several forms of ASD have been described, such as Asperger syndrome [3] or Kanner-type autism [4], revealing that ASD is a highly heterogeneous disorder, likely with multiple underlying causes. Intense scientific work has been performed in recent years to understand the potential origin of ASD, revealing that this disorder arises from both genetic and environmental factors, especially those influencing fetal and early-life development [5].

Although ASD has been shown to be highly heritable (recent estimates 38–54%), several meta-analyses have highlighted that nongenetic prenatal causes of ASD exist, opening the door for further studies to investigate such mechanisms [6]. Approximately 10% of ASD cases are linked to disorders of genetic etiology, such as fragile X syndrome, tuberous sclerosis, and Rett disorder. Supporting the idea of heterogeneity of ASD, single genetic mutations account for only 1-2% of ASD cases [7], with the majority of cases remaining idiopathic. Mutations identified by genetic studies have revealed that some affected genes are involved in brain development from* in utero* through infancy. Frequent aberrations in brain cytoarchitectural organization and neuronal connectivity have been observed in the brains of ASD patients, leading to the concept that ASD is a synaptopathy [8]. Genes involved in synapse formation or brain connectivity (e.g.,* fmr1*,* mecp2*,* shank3*,* tsc*,* neuroligin*, and* cntnap2*) have been repeatedly linked to ASD [9–11].

ASD brain transcriptome studies identify molecular abnormalities in synaptic and immune/microglia markers gene expression, with the former being downregulated and the latter upregulated [12]. Other genes related to inflammation (e.g.,* il-1raplp1*,* il-1r2*,* c4b*,* met*,* mch2*,* par2*,* mtor1*, and *μpar*) have been reported to be differentially expressed in ASD as well [13, 14]. This is of particular interest as the perinatal environment generating chronic neuroinflammatory processes leads to the rapid development of ASD in susceptible children [15]. Indeed, maternal inflammation linked to infection, autoimmunity, obesity, or gestational diabetes during pregnancy is associated with a higher risk of neurodevelopmental disorders, in particular ASD [16], as reviewed by Estes et al. [15]. Many experimental studies have linked maternal immune activation (MIA) in the pathogenesis of ASD with neuroinflammatory events in the developing brain as an important component of brain malformation [17, 18]. Experimental studies also revealed that MIA induces long-lasting changes in immune system activity and microbiota, which are believed to be involved in behavioral alterations in offspring [19, 20]. Interestingly, the host microbiota has been shown to modulate local immune responses in the brain [21], and conversely neuroinflammation can influence the microbiota composition [19]. In addition to the microbiota, nutrition is an important component of inflammatory regulation and nutritional deficiency could also be an important risk factor for ASD [22]. Recent animal studies have revealed that maternal nutritional statuses in n-3 polyunsaturated fatty acids (PUFAs), essential fatty acids with anti-inflammatory properties that are present in the brain [22–24], regulate microglia activity in the developing brain [25] and influence ASD-like behavioral disorders [26]. Here, we discuss evidence of neuroimmune dysregulation in patients with ASD, along with the epidemiological, clinical, and experimental studies implicating MIA, gut microbiota, and lipid nutrition as environmental factors that can lead to sustained neuroinflammation and contribute to the etiology of ASD. Understanding these risk factors could contribute to the development of novel nutritional strategies for therapeutic interventions in ASD.

## 2. Evidence of Neuroinflammatory Processes in Autism

**Figure 1:**
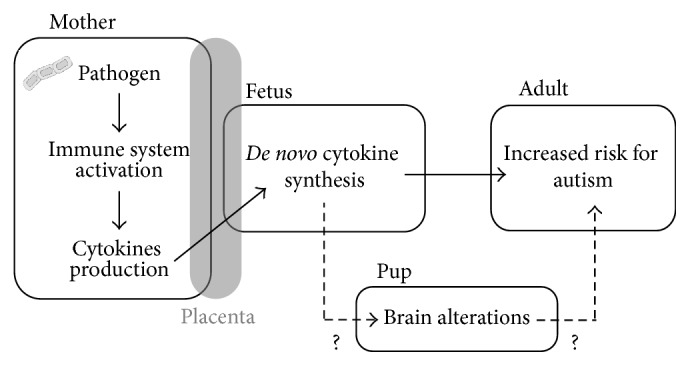
Association between prenatal infection and enhanced risk of neurodevelopmental disorders. During pregnancy, pathogens are thought to increase the risk of neurodevelopmental disorders in the offspring depending on the timing of infection and the magnitude of maternal immune response. Activation of the fetal immune system by* de novo* synthesis of cytokines sensitizes the brain to neurodevelopmental alterations. Interaction with other environmental and/or genetic factors also contributes to ASD etiology. Modeling prenatal immune activation represents a powerful tool to elucidate the relative contribution of these various factors for enhanced risk of ASD as well as other neurodevelopmental disorders.

Over the last 10 years, much evidence has accumulated pointing to inflammatory mechanisms as contributors to ASD, and intense research has been undertaken to determine exactly how immune dysregulation alters brain connectivity and function and plays a role in autism phenotypes [27] (Figure 1). The recent demonstration that microglia, the resident immune cells of the central nervous system (CNS), contribute not only to inflammatory events but also to neural development, has raised new hypotheses regarding their role in the etiology of autism. In addition to altered systemic immunity [28, 29], neuroinflammation has been observed in the brain of ASD patients. The presence of activated microglia has been reported in the dorsolateral prefrontal cortex of autistic patients [30]. Moreover, Positron Emission Tomography (PET) imaging studies have revealed an activation of microglia in other brain regions [31, 32]. Postmortem studies of individuals with ASD have also shown activation of microglia, as well as an increase in density [30, 33, 34]. Reinforcing the idea of immunological dysfunction in ASD [35–39], this activation of microglia is accompanied by increased expression of proinflammatory factors, such as cytokines and chemokines, in the brain and cerebrospinal fluid of ASD subjects [30, 34]. In particular, the proinflammatory cytokine IL-6 and the chemokines MCP-1 and RANTES have been reported in neonatal blood samples from ASD children [40]. Brain arginine vasopressin, which is released during inflammation and plays a role in social behavior in mammals, has also been associated with ASD [41] and is considered as a biomarker of the disease. Quinolinic acid and neopterin, which are activated by indoleamine 2,3-dioxygenase (IDO), an enzyme upregulated by inflammatory factors and involved in depression [42], are decreased in the cerebrospinal fluid (CSF) of ASD patients [43]. This may reflect an inadequacy or lack of maturation of the immune system. Despite the lack of evidence in humans that neuroinflammation plays a direct role in the pathogenesis of autism, research in animal models strongly suggests this to be the case. Deficits in microglial activity during brain development have been shown to be deleterious toward the formation of mature synapses, leading to an increase of immature synapses that could account for cognitive and ASD-like behavioral deficits [44, 45]. Therefore, in addition to genetic risk factors for inflammation, environmental factors leading to neuroinflammatory events are receiving more scrutiny in the etiology of autism. In this review, we will particularly focus on maternal immune activation (MIA), PUFAs, and microbiota as environmental risk factors that may participate in the etiology of ASD in combination to genetic risk factors.

## 3. Risk Factors for Neuroinflammation and Autism

### 3.1. Maternal Infection during Pregnancy and Autism Epidemiological Studies

Epidemiological studies strongly support a link between maternal infection and the development of ASD [18]. Compelling evidence supporting this hypothesis comes from a study on babies born from mothers exposed to the 1964 rubella pandemic. An increased incidence of children suffering from autistic disorders of 8–13% (versus 0.05% in controls) was found in this ecological cohort [46, 47]. Since then, ASD has been associated with numerous types of infectious agents, including not only viral, but also bacterial and parasitic infections [18]. Data collected from a Danish register of one million children born between 1980 and 2005 showed an association between infection-driven hospitalizations of pregnant women and an increased prevalence of ASD diagnoses in children. Interestingly, the time-window of infection is critical for the association with ASD and is different depending on the pathogen. The first trimester has been identified as critical for viral infections whereas bacterial infections during the second trimester have been associated with [16]. These observations suggest that the maternal immune effectors synthesized during infection rather than infection* per se* would be responsible for cerebral changes in the offspring leading to ASD. Furthermore, in addition to the temporal window of infection, the magnitude of inflammation (i.e., fever duration and hospitalization) is crucial for the prognosis of children born from infected mothers. Of note, recent evidence, showing that infection with Zika virus (ZIKV) during pregnancy induces major brain damage and microencephaly, has led to speculation on the role of this virus in developmental diseases such as ASD [48–50]. ZIKV has been shown to directly infect neural cells and promote their death but could also activate the immune system and in turn affect neuronal network-building in the fetus brain [51, 52].

One plausible mechanism supporting the association between maternal infection and ASD is cytokine production in the fetal brain in response to maternal inflammatory reaction [53]. Such cytokine expression may affect normal brain development in the offspring. In 2013, Zerbo et al. [54] showed that maternal fever during pregnancy is associated with ASD outcomes in the offspring while the risk of developing autism is reduced when mothers take antipyretic medications [54]. Moreover, mothers of children with ASD present higher blood levels of interferon gamma (IFN*γ*), IL-4, and IL-5 amid pregnancy [55]. Recent case-control studies have shed light on the positive correlation between proinflammatory cytokines levels in the amniotic fluid and occurrence of ASD [28, 56] (recently reviewed in Bilbo and Schwarz, 2012 [57]). Remarkably, IFN*γ* is critical for social behavior and frontocortical brain regions, a hallmark of ASD, as demonstrated in mice deficient in adaptive immunity, further reinforcing the link between social behavior and this cytokine [58]. Altogether, these associations give rise to the hypothesis that maternal immune activation (MIA) irremediably impacts the developing brain, which contributes to the etiology of autism [18, 59–61].

#### 3.1.1. Animal Models

The clinical evidence highlighting MIA as a risk factor for ASD has motivated the development of several animal models. In particular, infection of pregnant rodents with pathogens (virus and bacteria) relevant to human and activation of maternal immune system with viral or bacterial endotoxins in the absence of pathogen have been widely used (reviewed in Patterson, 2011 [18]). Interestingly, despite the fact that different molecular pathways are activated in these models, considerable overlaps have been found in behavioral impairment consistent with ASD symptoms.

#### 3.1.2. Active Viral/Bacterial Infections

Attempts to model prenatal infection in animals led to exposing pregnant rodents to the human influenza virus. Prenatally exposed offspring presented typical signs of altered neuronal migration [62], as well as astrogliosis [63], mimicking alterations found in ASD patients [61, 64]. In another prenatal infection study, Fatemi and colleagues reported increased expression of* Vldlr* and* Foxp2*, also consistent with data from human ASD patients [65]. Behavioral assessments of murine offspring are designed to mirror as closely as possible those used to observe ASD patients [66, 67]. Deficits in sensorimotor gating are typically assessed by a prepulse inhibition (PPI) paradigm, in which a weak prestimulus inhibits the reaction for a subsequent stronger startling stimulus. Patients suffering from ASD display deficits of prepulse inhibition as a manifestation of their general inability to filter out unnecessary information. This has been linked to abnormalities of sensorimotor gating. Adult offspring that had been exposed to influenza early in their gestation exhibit PPI deficits and altered exploratory and social behavior [68]. Recently, the influenza model was used in rhesus monkeys, an animal model more relevant for human brain development. Flu infection early in the third trimester leads to reduced volume of cortical grey matter, decreased white matter in the parietal cortex, and neuronal alterations. Such aberrations of brain development are all characteristic of ASD [5].

Bacterial infections have also been shown to increase the risk of developing autism [18]. Live bacterial infection models were developed in rodents by infecting dams with Group B* Streptococcus* (GBS), the most common human pathogen in fetal environments. When exposed to GBS during pregnancy, the offspring recapitulated numerous neurobiological and behavioral autistic-like symptoms. Moreover, a gender dichotomy appears in offspring, which is a cardinal feature of human ASD [69].

Taken together, findings obtained in animal models of viral and bacterial infections support the hypothesis of deleterious effects of a prenatal infection in ASD. Notably, viruses are never found in the brains of offspring, suggesting that the maternal immune response to infectious agents is more relevant than the agents themselves in the detrimental effects of prenatal immune challenges [68]. In fact, animal studies show that infectious agents do not usually reach fetal compartments; however, cytokines from the mother can still cross the placental barrier and stimulate* de novo* synthesis of cytokines in the fetal brain [70]. To test whether altered expression of maternal and/or fetal cytokines might play a role in linking maternal infection and development of autism, other models using immune-activating agents have been developed and are widely used in present-day studies.

#### 3.1.3. Viral/Bacterial Mimics

Viral and bacterial mimics activate the maternal immune system to induce cytokine release without any intervention of active viruses or bacteria with poly(I:C) and lipopolysaccharide (LPS) being the most studied. Poly(I:C) models have been very useful in deciphering the critical time-windows of infection relevant to ASD [71]. Poly(I:C) administration at midgestational time points (E9, E12.5) recapitulates ASD-like behavior in offspring, including decreased social behavior, ultrasonic vocalization deficits, repetitive behaviors, increased anxiety, and deficits in PPI [17, 72, 73]. Impaired ability to filter stimuli has been mostly associated with schizophrenia-like phenotypes, especially in rodents, but human adults suffering from ASD have similar sensorimotor gating deficits [74]. In rhesus monkeys, poly(I:C) injection during the first trimester leads to impaired social interaction, social attention, and repetitive behavior [75, 76]. Most of the behavioral impairments in offspring from mothers treated with poly(I:C) are observed with LPS [77]. Interestingly, late gestation administration of LPS triggered PPI deficits and social behavior alterations in offspring in adulthood [78, 79], while behavioral deficits appeared in infancy when mothers receive LPS at an early stage of gestation [80, 81]. Very low doses of LPS administered to rhesus monkeys at the end of gestation also induce PPI impairment in offspring [82]. Of note, LPS administration in mice pups at 14 days of postnatal age can also trigger behavioral deficits, which differ from adolescence to adulthood, with anxiety-like behavior appearing at adolescence, while depressive-like behavior develops during adulthood only [83]. Indeed, the exposition to viral or bacterial mimics during the whole brain developmental period seems to be critical for later life behavioral deficits classically observed in ASD.

Neurobiological changes induced by viral and bacterial mimics also share common features such as altered dopaminergic neurotransmission [70, 84, 85], altered myelin properties within frontostriatal-limbic circuits [86], an increase in GFAP-positive cells, hippocampal disorganization [87–89], and synaptic density turnover and transmission abnormalities [71]. Such impairment could be linked to alterations in developmental processes such as neuronal migration, establishment of neuronal layers, synaptogenesis, and synaptic pruning [90, 91]. Indeed, large number of reelin-expressing and newly born neurons are decreased in the hippocampus of poly(I:C)-treated pups whereas the amount of apoptotic cells is increased [92]. The decreased number of reelin-positive cells, together with GAD67- and parvalbumin-positive cells, is found in the developing hippocampus and prefrontal cortex of offspring from LPS-injected mothers [93–96]. Interestingly, early pregnancy administration of LPS increases spine density in the hippocampus of offspring during development but decreases it in adulthood [97], suggesting a transient developmental effect on spines close to the inflammatory response window. This is consistent with the observed activation of microglia, the brain's innate immune cell recently highlighted as key in developmental brain wiring [98, 99], in the brain of pups from poly(I:C)-injected dams [73]. Therefore, it appears that immune challenges during pregnancy lead to the impairment of structural development and wiring. This could be linked to altered expression of neuronal migration genes [100] or to defects in synaptic pruning and synaptogenesis with a plausible involvement of microglia [45].

Numerous studies have highlighted that developmental impairment triggered by inflammatory mimics could involve cytokines [72]. Indeed, poly(I:C) is a synthetic double-stranded RNA that induces inflammatory responses by binding to Toll-Like Receptor- (TLR-) 3 [101]. Like viral particles, poly(I:C) is a potent inducer of not only classical interleukins (e.g., IL-1*β* and IL-6) or TNF*α*, but also type 1 IFN (*α* and *β*). LPS, a gram-negative bacteria cell wall component, activates TLR4. Most of the cytokines produced in response to poly(I:C) or LPS are quite similar, except for type 1 IFN release, which is only elicited by poly(I:C). In addition, LPS treatment leads to a longer and larger release of IL-6 [58], a cytokine consistently increased in ASD patients [60, 102, 103]. Prenatal administration of poly(I:C) and LPS activates inflammatory response not only in mothers, but also in the fetus [76, 104, 105]. Overall, data using manipulation of cytokines have reported that IL-6 is essential for MIA-induced abnormalities in offspring's brain and behavior [17, 18, 20, 70, 106] and supports evidence from human ASD patients [17, 60, 102]. Recent data pointed that IL-17, a cytokine found in the blood of ASD children [107, 108] and of animal model of MIA [109], is involved in some symptoms of MIA-induced ASD-like behavior [110] providing additional data on the role of cytokines in fetal brain development.

In summary, MIA triggered by active pathogens or noninfectious endotoxins (poly(I:C) and LPS) administered during pregnancy recapitulates ASD-like behaviors and neurobiological alterations in offspring. MIA-induced long-term deficits depend on the stage of pregnancy that is targeted, in accordance with observational studies in humans [79, 92]. Animal models of MIA offer the opportunity to better understand the mechanisms underlying MIA and autism-like disorders to develop specific anti-inflammatory strategies to protect mothers at risk of having children with ASD.

#### 3.1.4. Interactions between ASD Risk Factor Genes and MIA

One important question arises from “inflammatory genes” × “inflammatory insults” as risk factors for autism. As previously described, MIA is an environmental risk factor for ASD that modulates the same inflammatory mediators identified as ASD susceptibility genes [111]. While many studies provide evidence for altered immune responses in patients with ASD [12, 111], recent transcriptome and protein interactome network analyses have revealed a direct link between genes implicated in ASD and immune signaling [112, 113]. Among the immunologic gene variants identified in ASD (e.g.,* mecp2*,* il-1*,* mhc*, and* c4*), many are expressed by microglia or modulate their activity, especially during brain development. Of note, the deletion of mGluR5, whose expression is decreased in the brains of ASD patients, increased the number of microglia in mice [114]. Indeed, the contribution of genetic factors and environmental insults targeting the immune status to ASD risk could be of particular importance during the developing period. Studies using transgenic mice with ASD-associated mutations reported developmental defects in these animals. However, to our knowledge, the interaction between MIA and immunity risk variants in ASD in humans or animal models has not yet been reported.

Several studies have reported that early-life inflammation has differential effects in patients or in transgenic mice with targeted mutation of genes identified in ASD. Early prenatal inflammation in mice (E9) has been shown to trigger some behavioral and neurobiological abnormalities in mice expressing the human mutation of* disc1* [115]. Autism-like behaviors such as sensorimotor gating deficiencies and impaired social behavior were modified by MIA depending on the type of* disc1* mutation. One-half of patients with tuberous sclerosis have been shown to develop ASD. In a mouse model of tuberous sclerosis (*tsc2* haploinsufficiency), maternal immune challenge led to impaired social behavior in adult offspring. Moreover, the authors found that seasonal flu activity in late gestation and TSC mutations increased the risk of ASD in offspring. TSC is involved in the mTOR pathway as well as other ASD-associated genes, for instance,* pten*,* eif4e*, or* fmr1* [15]. In another recent study, alterations in sensorimotor gating and attention processes were observed in the offspring of* Nurr1* heterozygous mice undergoing prenatal immune challenge [116]. In another study, a positive association was found between copy number variants in some hot spots for ASD pathology and maternal infection or fever during pregnancy [117, 118]. Epigenetic changes after maternal immune activation have also been observed in the offspring's brains, including abnormalities in histone acetylation in genes known to be involved in neurodevelopment [119]. Another work has identified hypomethylation of ASD-related genes such as Mecp2 after MIA [120]. Altogether, these data strongly suggest that mutations in immune or nonimmune genes and environmental inflammatory insults are key in ASD. However, further studies are needed to understand how these factors converge on common molecular networks during brain development.

### 3.2. Gut Microbiota and Autism

Emerging evidences suggest that the microbiome plays an important role not only in immunity but also in neurodevelopmental disorders such as autism [19, 20]. Bacteria within the gut are complex ecosystems which produce metabolites, such as short-chain fatty acids (SCFAs), vitamins, and antimicrobial peptides [121]. The gut microbiota and its metabolites participate to the body physiology, including the brain [122], while microbiota alterations, often referred to as dysbiosis, participate to numerous pathologies, including neuropsychiatric disorder. Importantly, food composition influences gut microbiota composition and very recent data obtained in rodents causally linked maternal diet, gut microbial imbalance, and neurodevelopmental disorders [123]. Among the pathways through which gut microbiota influences brain functions, the immune system is particularly relevant to neuroinflammation and ASD [20].

#### 3.2.1. Epidemiological Studies

Gut-brain interactions are now recognized to play a major role in neurodevelopment and in regulating behavior. In fact, ASD subjects often suffer from gastrointestinal distress [124], a comorbid factor for autism [125]. Gastrointestinal features include chronic abdominal pain and alterations in bowel habits, leading to questions about the nutritional status and the diet quality of children with ASD [125, 126]. Often, gastrointestinal symptoms remain mostly untreated and can give rise to behavioral alterations. Microbiome-related factors may also be responsible for increases in ASD prevalence [127]. Dysbiosis has been found in children with ASD compared to healthy controls [128, 129]. Gastrointestinal microflora is dysregulated in late onset autistic children [130], leading to alterations of microbial species density and variations of bacterial metabolites in feces and urine [131]. Studies have shown that the Clostridia species is consistently highly represented in feces from autistic children [129, 132]. There is also a greater abundance of* Bacteroides* and Firmicutes in severe ASD [130, 133]. Clostridia toxins are known to affect neurotransmitter functions that can possibly result in neurobehavioral changes. Dysregulated activity of the autonomic nervous system, associated with anxiety and stress-responsiveness, may also play a role in increased intestinal epithelial permeability in ASD subjects [134], leading to observed behavioral changes. Altered intestinal permeability could represent a possible explanation for behavioral abnormalities observed in ASD, as immune molecules or products of diverse microbial populations could more likely enter the blood circulation and affect the brain. Conversely, antibiotic therapy using vancomycin during a short period improved behavior [135]. Dysregulated gut-brain communications, in addition to genetic heritability, could account for some of the extreme diversity seen across wide spectrum for autism, depending on the severity of alterations of microbial communities.

#### 3.2.2. Animal Models

Only a few studies have shown mechanistic connections between alterations of the gut microbiota and behavioral changes observed in ASD patients. Rodent models are useful for examining these interactions and to discover new targets from diet patterns to therapeutic treatment using probiotics instead of antibiotics.

Rodent models of ASD have been used to determine a link between alterations of the gut microbiome, associated changes in microbial factors, and their implication in behavioral changes observed in autistic-like behavior [136]. These changes were rapidly reversed by the use of probiotics in an MIA model [20]. Clostridia and* Bacteroides* species were the primary drivers of these microbiota differences. Offspring from an MIA model that received* Bacteroides fragilis* as a probiotic significantly recovered the abundance of some taxis. Moreover,* B. fragilis* dramatically attenuated altered behavior observed in offspring including communication, repetitive behaviors, and reduced anxiety. Animals subjected to valproic acid exposure* in utero*, a mouse model of ASD, show disturbed social interactions and increased expression of neuroinflammatory markers alongside intestinal inflammation [136]. Prenatal exposure to valproic acid has a transgenerational impact on the gut microbiota as observed by increased levels of short-chain fatty acids (SCFAs) like butyrate in the caecum of offspring [136]. Interestingly, SCFAs are considered neuroactive metabolites as they can cross the blood-brain barrier and modulate CNS function and behavior [137–139]. Interestingly, prenatal administration of propionic acid, a SCFA byproduct of enteric bacteria found in ASD subjects [140], triggers some of the ASD-like behavior [141]. Notably, propionic acid intracerebral administration activates microglia, suggesting a role of this SCFA in neuroinflammation [139]. Because the maternal transmission of immune factors induces specific changes in the gut microbiome, it could therefore affect the neurometabolites available to the offspring that could potentially lead to autistic-like behaviors or alterations of the gut epithelium. Further studies are needed to better understand whether changes in the gut microbiota of children could be a risk factor for dysbiosis, neuroinflammatory processes, and ASD.

#### 3.2.3. Interactions between ASD Risk Factor Genes and Gut Microbiota

Abnormalities in immunity could be closely linked to the gut microbiota and dysbiosis in ASD. The gut microbiota stimulates both nonspecific and specific immunity in the first years of age [142] and has been recently suggested to regulate microglia activity [21]. After birth, the low-grade inflammation, although generally beneficial, triggered by the continuous immune stimulation provided by the gut microbiota [143] could be detrimental in children at risk for ASD because of genetic synaptic dysfunction. However, such a link has been poorly studied. Recently, transgenic mice with a defect in inflammasome/IL-1*β* production (i.e., caspase 1 KO mice) have been shown to have a different microbiota composition than wild-type mice, together with depressive-like behavior, suggesting that behavioral impairment linked to dysbiosis requires inflammasome activity [144, 145]. Whether a specific interaction between genes identified as risk factors for autism and dysbiosis/microbiota changes exists in patients with ASD is unknown. Further clinical and fundamental research on this issue is warranted.

### 3.3. Dietary N-3 Polyunsaturated Fatty Acids and Autism

**Figure 2:**
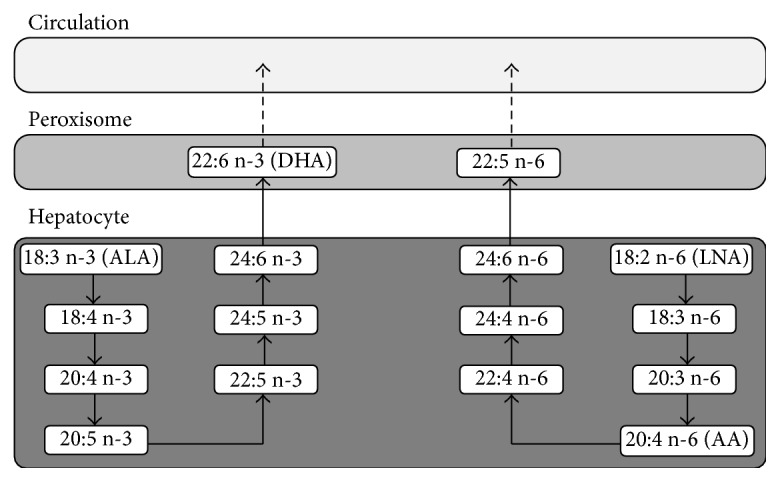
Synthesis of PUFAs in the liver. Precursors of n-3 and n-6 polyunsaturated fatty acid (PUFA) *α*-linolenic (ALA; 18:3 n-3) and linoleic acid (LNA; 18:2 n-6) can be desaturated and elongated. This leads to the synthesis of long-chain PUFAs, including docosahexaenoic acid (DHA; 22:6 n-3), but also arachidonic acid (AA; 20:4 n-6) which are carried into the blood as free forms or lipoproteins. Both, n-3 and n-6 long-chain PUFAs, compete for their synthesis (for desaturation and elongation), meaning that PUFAs intake significantly impacts their cerebral incorporation level.

Several studies have highlighted the fundamental role of lipids in neuronal processes and immune modulation, which are implicated in ASD. In particular, polyunsaturated fatty acids (PUFAs) are essential fatty acids required for brain development and maturation [22]. Because they need to be provided by alimentation (Figure 2), deficiencies or imbalances in these nutrients, both precursors and long chains strongly affect brain function, not only during development, but also throughout life and especially during periods of neuroinflammation. Recent evidence suggests that n-3 PUFA homeostasis may be altered in ASD, either as a result of nutritional imbalance or genetic defect [146].

#### 3.3.1. Epidemiological Studies

Total n-3 PUFAs in the plasma of autistic children are decreased without any changes in the n-6 PUFAs family [147, 148]. A positive association between anti-myelin basic protein (MBP) antibodies and low levels of the main n-3 PUFA found in the brain (docosahexaenoic acid, DHA) has been reported in autistic children [149]. Parental health questionnaires and red blood cell (RBC) fatty acid measurements have highlighted a decrease in DHA and total n-3 PUFAs in both autistic and Asperger patients. More recently, Al-Farsi and colleagues reported lower consumption of DHA foodstuff and a concomitant decrease in DHA levels in the plasma of children with ASD [150]. A case-control study in California measured fatty acids in the blood of 153 autistic children and 97 controls and showed that DHA is decreased in the phosphatidylethanolamine (PE) [151]. Another case-control study in Saudi Arabia showed altered phospholipid and fatty acid profiles in ASD patients [152]. Consistent with the idea of impaired PUFAs cerebral level and metabolism, Brigandi and colleagues uncovered a massive decrease in AA and DHA. They also found an increase in proinflammatory derivative Prostaglandin E2 in a subset of patients [153]. Interestingly, gene expression of FABP3, FABP5, and FABP7 has been shown to be modulated in psychiatric illnesses such as schizophrenia and ASD [154]. In the brains of ASD patients, FABP7, which binds DHA preferentially, was upregulated in both the frontal and parietal cortex [155]. As in schizophrenic patients, PUFA distribution and metabolism are markedly altered in ASD patients. Six weeks of DHA and eicosapentaenoic acid (EPA) supplementation in children with autism led to improvement of symptoms, especially stereotypy and hyperactivity [156]. A 12-week n-3 fatty acid dietary supplementation also led to the improvement of hyperactivity in autistic children [157]. Another study using a DHA, EPA, and AA dietary supplementation for 3 weeks in autistic children reported improved behavioral performance in two-thirds of children [158]. Recently, an open-label pilot study in Singapore found positive associations between blood fatty acid levels and changes in the core symptoms of ASD following a 12-week n-3 PUFA dietary supplementation [159]. However, several interventional studies with n-3 PUFAs failed to reproduce these beneficial effects [160–162]. Thus, larger cohorts and accurate ASD behavioral phenotypes are needed to clearly decipher the potential beneficial effects of n-3 PUFA dietary supplementation on behavioral deficits. In addition, the inflammatory status and/or the microbiota composition should be considered in interventional studies with n-3 PUFAs [15, 124].

#### 3.3.2. Animal Models

Some human-like ASD alterations were observed in preclinical models of n-3 PUFA dietary deficit. Developmental n-3 PUFA depletion in rodents led to decreased levels of serotonin in the prefrontal cortex, as observed in autistic children [163, 164]. Numerous studies on n-3 PUFA deficiency models revealed profound alterations in GABAergic, dopaminergic, and cholinergic neurotransmission [165–168]. Importantly, long-term dietary n-3 PUFA deficiency triggers the development of ASD-like behavioral impairment in rodents, including reduced PPI [169], social interactions [170–174], and increased anxiety [171–173, 175]. Conversely, some studies investigated the possible beneficial role of n-3 PUFA dietary supplementation at weaning in different mice models of ASD. In the Fmr1-KO mice model of autism, n-3 PUFA supplementation rescues not only social defects but also memory impairments and some neurobiological imbalance [26]. In a model of prenatal inflammation by poly(I:C), DHA supplementation improves social interactions, decreases repetitive behaviors, and normalizes IL-6 levels after immune challenge [176]. A recent study on an early MIA model showed that n-3 PUFA-enriched diet alleviates ASD-like symptoms, altered GAD67 protein levels, metabolic changes, and PPI deficits [177]. As n-3 PUFAs potently regulate neuroinflammatory processes, microglia activity, and synaptic plasticity [24, 174, 178], their beneficial effects could be linked to their action on neuroinflammatory processes in the developing brain. Interestingly, n-3 PUFAs modify the gut microbiota composition, but their effect in ASD-like behavior has not yet been unraveled [179].

Taken together, both studies in humans and animals identify long-chain PUFAs, especially those from the n-3 series, as interesting candidates in curative strategies due to their ability to counteract some ASD-like symptoms and ameliorate inflammation. Several studies have also shown their ability to modulate the microbiota and vice versa. Indeed, one study reports that SCFA propionic acid administered into the brain of rats alters lipid metabolism, in particular the one of PUFAs [180]. According to a recent report, n-3 PUFA deficiency induces dysbiosis, with increased numbers of potential pathobionts, including bacteria from the Enterobacteriaceae family [181]. Conversely, n-3 PUFA supplementation prevents the bloom of Enterobacteriaceae, as well as the translocation of bacteria into the submucosal region, and instead promotes the enrichment of* Lactobacillus* and* Bifidobacterium* species [181, 182]. Using a genetic model of n-3 PUFA supplementation (Fat-1), Kaliannan et al. demonstrated that elevated n-3 PUFA levels enhance intestinal production and secretion of intestinal alkaline phosphatase (IAP), which induces changes in the gut bacteria composition, resulting in decreased LPS production and gut permeability and, ultimately, in reduced metabolic endotoxemia and inflammation [183]. N-3 PUFA deficiency during development (over gestation and lactation) also alters the normal trajectory of intestinal microbe establishment in the intestine of offspring, with lowered bacterial density, a decreased ratio of Firmicutes to Bacteroidetes, and a decrease in several other dominant microbes [184]. These data suggest that n-3 PUFA levels modulate microbiota composition and activity during development. However, more results are needed to unravel the underlying mechanisms.

N-3 PUFAs are likely to be taken up in large amounts by the brain during the end of gestation and the first month of life [185, 186]. The use of n-3 PUFA supplementation, especially during pregnancy and lactation, could help prevent ASD in children at risk. In this context, developmental animal studies giving n-3 PUFA supplementation from conception might be a fruitful line of investigation.

#### 3.3.3. Interactions between ASD Risk Factor Genes and Dietary PUFAs

Genetic interactions and PUFAs content have been poorly studied in ASD. However, some links exist between lipid metabolism gene alleles, PUFA metabolism, and brain diseases. Indeed, the APOE4 allele, which is a well-known genetic risk factor of Alzheimer's disease, is involved in disrupted PUFA metabolism with a shift to long-chain n-3 PUFA oxidation [187, 188]. Genetic variability in* fads* (desaturases involved in the metabolization of PUFAs) is involved in the bioavailability of long-chain PUFAs AA and DHA to the brain, as well as brain development and cognitive impairment [189–192]. Polymorphism of several genes involved in PUFA metabolism or inflammation is crucial in the efficacy of dietary n-3 PUFA supplementation on inflammation and triglyceride blood level [193, 194]. The relationship between PUFA metabolism genes, inflammation, and efficacy of n-3 PUFA dietary supplementation remains to be determined. This is of particular importance as concentration and expression of phospholipase A2, a phospholipase at the cross of PUFA metabolism and inflammation, are higher in ASD patients but are reduced by dietary supplementation with EPA [195–197]. N-3 PUFAs potently regulate not only neuroinflammatory pathways [24, 178, 198] but also synaptic plasticity [25, 173, 199–201]. These properties could be of high interest in correcting synaptic defects linked to genetic risk factors. Indeed, dietary n-3 PUFA supplementation rescues social behavioral impairment and neuroinflammation in a mouse model of fragile X syndrome [26].

## 4. Conclusion

**Figure 3:**
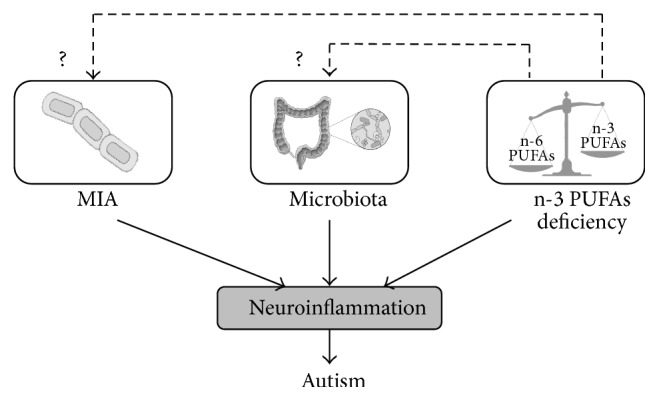
Environmental factors influencing neuroinflammation in autism. Early inflammation in the brain is a well-recognized risk factor for autism. Neuroinflammation is a process influenced by environmental factors such as MIA, microbiota, and n-3 PUFAs deficiency. However, crosstalks between these factors can make the situation increasingly complex. For instance, insufficient dietary n-3 PUFAs intake unavoidably impacts microbiota composition as well as MIA immunoreactivity possibly potentiating the proinflammatory response. This, in turn, can lead to increased risks for autism.

The pathogenesis of ASD is linked to maternal immune activation-triggered neuroinflammatory events in the developing brain of offspring, potentially in association with dysbiosis during pregnancy and/or infancy. The dysregulation of these components during early development leads to brain malformation and alterations that can be imprinted until adulthood. Thus, elucidating the brain-microbiota axis is critical for finding more effective strategies to prevent or treat ASD. In particular, nutritional interventions, especially those taking advantage of the anti-inflammatory properties of n-3 PUFAs, are promising candidates, as they would potentially modulate both neuroinflammatory components and microbiota dysbiosis in ASD (Figure 3). Further studies are therefore needed to decipher the mechanisms underlying the beneficial effect of n-3 PUFA diets in ASD.
